# ROS-Mediated Signalling in Bacteria: Zinc-Containing Cys-X-X-Cys Redox Centres and Iron-Based Oxidative Stress

**DOI:** 10.1155/2012/605905

**Published:** 2011-09-29

**Authors:** Darío Ortiz de Orué Lucana, Ina Wedderhoff, Matthew R. Groves

**Affiliations:** ^1^Department of Applied Genetics of Microorganisms, Faculty of Biology and Chemistry, University of Osnabrueck, Barbarastr. 13, 49069 Osnabrueck, Germany; ^2^European Molecular Biology Laboratory (EMBL), EMBL Hamburg Outstation, c/o DESY, Building 25A, Notkestrasse 85, 22603 Hamburg, Germany

## Abstract

Bacteria are permanently in contact with reactive oxygen species (ROS), both over the course of their life cycle as well that present in their environment. These species cause damage to proteins, lipids, and nucleotides, negatively impacting the organism. To detect these ROS molecules and to stimulate the expression of proteins involved in antioxidative stress response, bacteria use a number of different protein-based regulatory and sensory systems. ROS-based stress detection mechanisms induce posttranslational modifications, resulting in overall conformational and structural changes within sensory proteins. The subsequent structural rearrangements result in changes of protein activity, which lead to regulated and appropriate response on the transcriptional level. Many bacterial enzymes and regulatory proteins possess a conserved signature, the zinc-containing redox centre Cys-X-X-Cys in which a disulfide bridge is formed upon oxidative stress. Other metal-dependent oxidative modifications of amino acid side-chains (dityrosines, 2-oxo-histidines, or carbonylation) also modulate the activity of redox-sensitive proteins. Using molecular biology, biochemistry, biophysical, and structure biology tools, molecular mechanisms involved in sensing and response to oxidative stress have been elucidated in detail. In this review, we analyze some examples of bacterial redox-sensing proteins involved in antioxidative stress response and focus further on the currently known molecular mechanism of function.

## 1. Introduction

Interference in the balance between the environmental production of reactive oxygen species (ROS), including hydroxyl radicals (^•^OH) and hydrogen peroxide (H_2_O_2_), and the ability of biological systems to readily detect and detoxify them, or repair the resulting damage, are defined as oxidative stress. Highly reactive radicals cause the oxidative damage of different macromolecules—proteins, DNA, and lipids—leading to loss of function, an increased rate of mutagenesis, and ultimately cell death. In humans, for example, oxidative stress is involved in many diseases, such as rheumatoid arthritis, autoinflammatory diseases, neurodegenerative diseases, and cancer [1, 2]. However, the production of some ROS (e.g., ^•^OH) can also be beneficial, as they are used by the human immune system to attack and kill pathogens, such as the production of ROS by macrophages. Additionally, H_2_O_2_ is an important signalling molecule that participates in redox signalling [3].

Sensing of ROS-mediated signals also plays a crucial role in the biology of microorganisms. Bacteria, for example, are in continuous contact with ROS generated both endogenously, as a product of aerobic metabolism, or exogenously during ionizing (*γ*) and nonionizing (UV) irradiation leading to the production of a number of radical and peroxide species through the ionization of intracellular water. Industrial contaminants that are widespread in soils and on the surfaces of plants are also sources of ROS. 

Iron is earth's fourth most abundant metal, after oxygen, silicon, and aluminium. Its relevance for bacterial cells is emphasized by the fact that it is involved in a wide range of biological processes, including photosynthesis, N_2_ fixation, H_2_ production and consumption, respiration, oxygen transport, and gene regulation [4, 5]. However, in the presence of oxygen, ferrous ions frequently result in oxidative stress through the generation of hydroxyl radicals *via* the Fenton reaction (Fe^2+^ + H_2_O_2_→Fe^3+^ + ^•^OH + ^−^OH). Therefore, bacteria have developed a variety of different mechanisms to ensure that iron is sufficiently accessible as well as being maintained in a nontoxic form [4]. They possess high-affinity iron transport systems (i.e., siderophores and membrane iron transporters) that enable iron to be scavenged. Intracellular iron can be stored in protein complexes (i.e., in Dps and ferritins). Thus, the homeostasis of these ions is tightly regulated so that their intracellular concentrations do not reach toxic levels. Previously, it has also been suggested that the production of hydroxyl radicals is induced by bactericidal antibiotics to kill bacteria, in which iron ions and the Fenton reaction play a role [6], or during a wide range of plant or human pathogen interactions. Moreover, ROS can also be produced during the degradation of natural existing biopolymers (cellulose, chitin, or xylan) by microorganisms [7]. To reduce the hazardous effects of iron-based production of ^•^OH, bacteria produce proteins with an enzymatic activity to degrade ROS (i.e., superoxide dismutases, catalases, peroxidases, and alkylhydroperoxide reductases), other small redox proteins (thioredoxins and glutaredoxins) as well as low molecular-weight thiols (glutathione and mycothiol) [8, 9]. All these cell components contribute in maintaining a reducing environment both in the cell and in controlling the extent of the oxidative burst. 

In analogy to Fe(II) ions, other transition metals ions [i.e., Cu(I), Co(II), Mn(II), Ti(III), or Cr(V)] are closely linked with the production of free radicals in cells [10]. Although these metal ions can be hazardous for living organisms, they also serve as signal mediators in signalling cascades. Another metal with high biological relevance is zinc. It is an essential trace element that is localized in the active center or in a structurally important site of many bacterial proteins [11]. Zinc is a cofactor for more than 300 enzymes (i.e., superoxide dismutase and alcohol dehydrogenase). It is also a structural element of at least 40 protein classes (i.e., RNA polymerase and tRNA synthetases). Additionally, zinc can protect sulfhydryl groups from free radicals and inhibits free radical formation by competing with redox-active metals such as iron [12, 13]. Binding of zinc as well as metal-catalyzed oxidation in proteins is closely related with the presence of redox-active cysteine residues (e.g., within Cys-X-X-Cys motifs) and other metal-sensing amino acids. Moreover, bacterial redox sensory proteins can act either as single transcription regulators (e.g., FurS or Irr) displaying a sensory and response domain within itself [14, 15], or as a part of multicomponent systems (e.g., HbpS or ChrS) in which sensing and response are distributed among each protein component [16–18]. 

In bacteria, there are a high number of redox sensory proteins that show different mechanism of function. Here, we will give an overview of them and focus further on signalling pathways in which redox-active cysteines as well as iron ions are involved.

## 2. Zinc-Containing Cys-X-X-Cys Motifs as Sensor Centres

**Figure 1:**
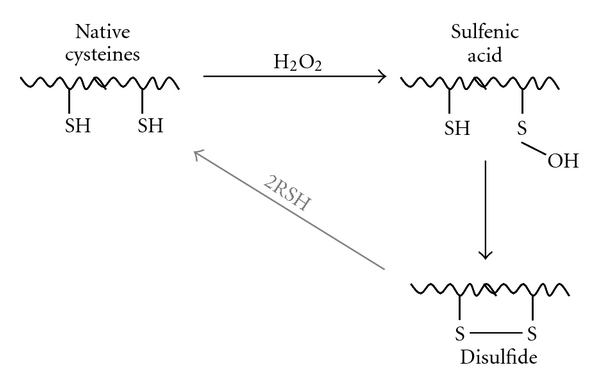
Formation of intramolecular disulfide bonds (S–S). Oxidation of a cysteine thiol by H_2_O_2_ yields a sulfenic acid residue that can undergo reaction with a neighbouring “back door” cysteine thiol to generate a disulfide linkage (S–S). S–S bonds can overtime be returned to the native SH state by reactions with biological thiols (RSH). This picture was adapted from [21].

Cysteine residues (Cys) in proteins are prominent targets for protein oxidation, as they easily react with H_2_O_2_ and free radicals. Oxidation of Cys by H_2_O_2_ involves nucleophilic attack of the cysteinyl thiol group on the electrophilic center of H_2_O_2_. Deprotonation of the Cys thiol group to generate the thiolate anion increases its nucleophilicity, and hence reactivity towards H_2_O_2_. These reactions are chemically highly complex and can lead to different sulfur oxidation states, including thiols, sulfenic and sulfinic acids, thiyl radicals, disulfide S-oxides, or disulfides [19]. Disulfides can be generated between two Cys either intra- or intermolecularly (Figure 1). The sensitivity of Cys thiol groups to oxidation provides them with redox sensitivity, and hence the ability to sense redox status. The molecular environment of the redox sensitive Cys also modulates the sensing mechanism. For example, the thiolate anion can be stabilised by proximity to hydrogen bond donors, basic residues, and metal ions [20].

In this review, we will focus on intramolecular disulfides that are formed within Cys-X-X-Cys motifs—(X: any amino acid)—a motif that is widespread in bacterial sensor proteins (Table 1). In addition to redox-sensing properties, these motifs are often involved in zinc binding (i.e., in FurS, Hsp33, RsrA, RslA, Trx2, and SbcC) (Table 1), and in the stabilization of protein domains that are crucial for function. It can be expected that the molecular environment of Cys is in this case also important for zinc binding.

Table 1 comprises two different types of Cys-X-X-Cys-containing proteins. Proteins belonging to the first group exhibit enzymatic function (Hsp33, ResA, DsbA, SbcC, cytochrome c, Trx2, AhpF/AhpC, CopA, and HypA). For example, under reducing conditions, the chaperone Hsp33 binds a single zinc ion through its C-terminal Cys-X-Cys-X_6_-Cys-X-X-Cys motif. Upon oxidative stress, the four Cys residues form intramolecular disulfide bridges resulting in release of zinc accompanied by considerable conformational changes that lead to destabilization of its C-terminus [22]. As a consequence, Hsp33 dimerises and acquires a chaperone function to prevent protein aggregation [23]. Similarly, in the DNA repair protein SbcC, two monomers are linked *via* Cys-X-X-Cys motifs and a zinc ion, forming a functional zinc bridge that is important for SbcC exonuclease activity [24]. ResA—an extracellular low potential thiol-disulfide oxidoreductase—was shown to maintain Cys residues of cytochrome *c* in their reduced form. The thiolate species within the heme-binding motif (Cys-X-X-Cys-His) of apo-cytochrome *c* are required for covalent attachment of heme [25]. Contrary to the other Cys-X-X-Cys-containing proteins described here, ResA does not bind zinc through this motif. Other proteins with this signature and lacking zinc are listed in Table 1 but are not within the focus of this review.

The second group of Cys-X-X-Cys-containing proteins is involved in transcriptional regulation and can be subdivided into two subtypes: proteins that either directly control transcription (i.e., WhiB3, FurS, CatR, SoxR, and SurR) or require further regulatory components (i.e., Spx, RsrA, RslA, and RshA). 

The redox-dependent Cys-X-X-Cys-containing transcriptional regulator WhiB3, from *Mycobacterium tuberculosis,* has been shown to sense the intracellular redox state in the cell, and to be required for the production of virulence polyketides, including polyacyltrehaloses (PAT), sulfolipids (SL-1), *di-o-acyl-trehaloses* (DATs) and trehalose dimycolates (TDMs). WhiB3 directly regulates the expression of the following genes in a redox-dependent manner: *pks3* (encoding a polyketide *beta*-ketoacyl synthase involved in PAT and DAT anabolism), *pks2* (encoding a polyketide synthase involved in SL-1 anabolism), and *fbpA* (encoding a fibronectin-binding protein for TDMs production) [26]. WhiB3 contains an iron-sulfur cluster [4Fe-4S]^ +^ that under aerobic conditions is oxidized to [4Fe-4S]^2+^. Further oxidative conversions result in complete loss of this cluster [27]. Unlike the superoxide stress sensor SoxR, the redox state of the WhiB3 iron-sulfur cluster does not modify the DNA binding affinity to target gene promoters, but rather the redox state of the cysteine residues is critical for DNA binding. It was shown that, while the reduction of Cys in WhiB3 abolishes its DNA binding activity, oxidation induces it. It is noteworthy to mention that reduction and oxidation in WhiB3 are reversible processes.

In the case of the zinc-containing transcriptional repressor FurS from *Streptomyces reticuli*, it was biochemically and spectrophotometrically shown that H_2_O_2_-mediated disulfide bond formation between Cys93 and Cys96 in FurS is accompanied by the release of the bound zinc ion. This leads to conformational changes in FurS, resulting in a loss of FurS binding to its own DNA operator sequence within the regulatory region of the target operon, *furS*-*cpeB*. As a result, the expression of this operon is no longer repressed, leading to an increase in production of FurS and the mycelium-associated catalase-peroxidase CpeB. This enhanced expression in turn detoxifies the surrounding environment of hazardous H_2_O_2_ [9, 14]. 

**Figure 2:**
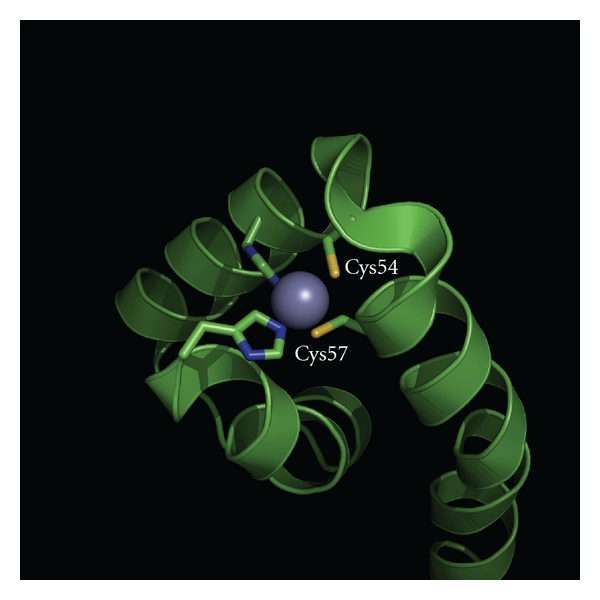
An image showing the redox-sensing cysteine residues (Cys54 and Cys57) within the crystal structure of RslA from *Mycobacterium tuberculosis* (PDB: 3HUG). The coordinated zinc ion (shown as a violet ball) strongly contributes to the overall fold stability.

Proteins with Cys-X-X-Cys motifs are also indirectly involved in transcriptional regulation, by interacting with additional components. Reversible disulfide formation can regulate the activity of the sigma factor R (*σ* ^R^) of *Streptomyces coelicolor* A3(2). Here, the thiol-specific oxidant diamide induces *σ* ^R^-mediated transcription of >30 genes, including those encoding thioredoxins and thioredoxin reductases that are involved in antioxidative stress response. However, *σ* ^R^ does not contain any Cys and *in vitro* studies have demonstrated that its transcriptional activity is not dependent on oxidative stress. This strongly suggests that an additional component must act as the oxidative stress sensor [28]. Indeed, researchers have characterised the protein RsrA, which interacts with *σ* ^R^ and regulates its transcriptional activity in a oxidative stress-dependet manner [29]. RsrA is a zinc-containing and redox-sensitive antisigma factor that possesses seven Cys, two of which (Cys41 and Cys44) are located within a Cys-X-X-Cys motif that is important for activity. Under oxidising conditions, a disulfide bond is formed in RsrA, and this oxidized state cannot bind *σ* ^R^ or inhibit its transcriptional activity. Oxidised RsrA can be re-reduced by thioredoxin and in the thiol-reduced state RsrA reassociates with *σ* ^R^ and blocks its transcriptional activity. The redox sensing Cys in RsrA also coordinate zinc ions that provide the protein with a higher structural stability, strengthening its interaction with *σ* ^R^. Upon redox stress and induction of disulfide bond formation, zinc is released and RsrA undergoes conformational changes, generating a structure that does not bind *σ* ^R^[30]. Structural studies of an RsrA homolog, RslA (Table 1) from *Mycobacterium tuberculosis *revealed that the redox sensing and zinc binding Cys (Cys54 and Cys57) are closely located (~3, Å; Figure 2) to each other and are solvent exposed in the complex, thus, providing a structural basis for the redox sensitivity of RslA [31]. The solvent exposed state of Cys within the Cys-X-X-Cys motif is also an essential feature in redox sensing by the heat-shock protein Hsp33 (Table 1; PDB: 1VQ0) from *Escherichia coli* [22]. 

## 3. Iron-Based Oxidative Stress

**Figure 3:**
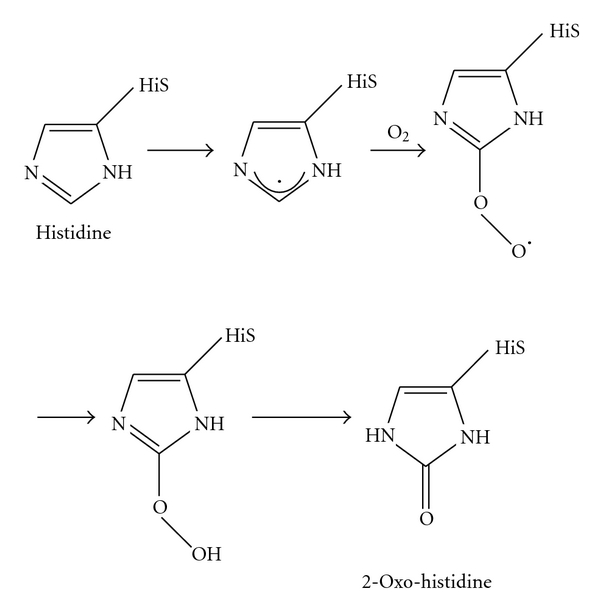
Metal-catalyzed oxidation of histidine. The exact chemical mechanism of the iron-mediated formation of 2-Oxo-histidine remains unclear. This picture was adapted from [34].

To assure that iron is sufficiently accessible as well as being maintained in a nontoxic form, bacteria have developed a variety of protein-based protection mechanism. They employ iron-sensing regulatory proteins (i.e., PerR and HbpS-SenS-SenR; see also Table 2) that control the expression of proteins (i.e., H_2_O_2_-degrading enzymes such as catalases) blocking iron-dependent damage. Upon sensing of iron-based stress, these proteins undergo different oxidative modifications, including oxidation of histidines (Figure 3), dityrosine formation, or carbonylation. 

Table 2 lists different iron-sensing proteins that mostly control redox stress resistance systems. They can directly regulate the transcription of target genes by DNA binding to regulatory regions (i.e., Fur, DtxR, RirA, Irr, PerR, DmdR1, IdeR, SirR, FNR, and TroR) or utilize further regulatory elements (HbpS-SenS-SenR; PmrA-PmrB, ChrS-ChrA, and FecA-FecR-FecI) that mediate the adaptive response. 

Iron-dependent gene regulation has been demonstrated for the diphtheria toxin repressor (DtxR) from *Corynebacterium diphtheriae* and *Corynebacterium glutamicum* [35]. Binding of divalent iron ions to the global regulator DtxR results in the transcriptional repression of a number of genes required for iron uptake (i.e., *htaA* coding for a secreted iron acquisition and transport protein), as well as for usage and storage (i.e., *hmuO* coding for a heme oxygenase and *ftn* coding for a ferritin-like protein). Mutational analyses have additionally demonstrated that DtxR is also involved in the iron-dependent expression of DNA-protecting proteins, including Dps-like proteins that bind iron and DNA. 

In *Bacillus subtilis,* the peroxide resistance regulator PerR has been shown to sense metal dependent as well as H_2_O_2_-based oxidative stress, and to regulate the corresponding adaptive response [36]. The metalloprotein PerR is a small dimeric protein that coordinates two metal ions per monomer. One binding site binds a zinc ion, whereas the second one a regulatory metal, either iron or manganese [37, 38]. *In vivo*, the regulatory metal is required for repression of the transcription of target genes, including those ones encoding a catalase, alkylhydroperoxide reductase, and the Dps-like DNA-binding protein MrgA. Under H_2_O_2_-based oxidative stress conditions, Fe^2+^ catalyzes the oxidation of histidines (either His37 or His91) in the regulatory binding site of PerR. This results in Fe^2+^ release and subsequent derepression of PerR target genes. Analysis of electron maps density in the crystal structure of the oxidised PerR protein (PerR-Zn-ox) showed the presence of a 2-oxo-histidine residue at position 37. Further, MALDI-TOF and tandem ESI-MS studies additionally revealed the oxidation of His91 within Per-Zn-ox [36, 38, 39]. It was concluded that the structural conformation of PerR is dependent on the oxidation state of the regulatory site. 

Oxidative modifications have also been shown to regulate the activity of the redox sensor and heme-binding protein HbpS from *Streptomyces reticuli*. HbpS is extracellularly located and interacts with the membrane-embedded histidine autokinase SenS from the two-component system SenS SenR [40, 41]. Analyses of the crystal structure of HbpS combined with size exclusion chromatography and static light scattering [42–44] allowed the identification of HbpS as an octamer. Further studies demonstrated that the octameric assembly in HbpS is required for an efficient interaction with SenS [43]. Phosphorylation analyses also revealed that under nonoxidative stress conditions HbpS inhibits the autophosphorylation of SenS, whereas oxidative stress induces the HbpS-mediated activation of SenS [16]. After autophosphorylation at a conserved histidine, SenS transfers the phosphate group to its cognate response regulator SenR, resulting in SenR~P. SenS acts also as a phosphatase of SenR~P. The unphosphorylated form of SenR binds to specific sites upstream of the *furS*-*cpeB* operon (encoding for the redox regulator FurS and the mycelia-associated catalase-peroxidase CpeB), leading to its transcriptional repression. Once SenR has been phosphorylated, it loses the ability to bind to this operator, leading to a de-repression of the *furS*-*cpeB* transcription. In addition, SenR~P has been shown to activate the transcription of *hbpS.* Comparative physiological analyses have demonstrated that the presence of HbpS and SenS-SenR provides *S. reticuli* with a defence system against redox-stressing conditions [40, 41].

**Figure 4:**
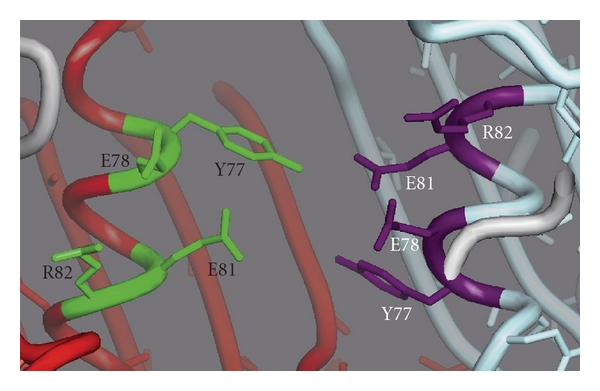
An image showing the arrangement of the internal EXXE motif between two subunits (red and turquoise chain, respectively) in the octameric HbpS (PDB: 3FPV). The amino acids Tyr77 (Y77), Glu78 (E78), Glu81 (E81), and Arg82 (R82) are indicated.

It was proposed that the switching of HbpS from its inhibitor to activator state of SenS autophosphorylation under oxidative stress conditions is controlled by conformational changes in HbpS. Indeed, CD spectroscopy as well as FRET analyses revealed that after iron-mediated oxidative stress HbpS undergoes secondary structure and overall intrinsic conformational changes, which are accompanied by oxidative modifications (i.e., carbonylation and dityrosine formation). While the sites of carbonylation have not yet been determined, the tyrosine residues participating in dityrosine formation have been identified as Tyr77. These residues are localized in the interface of HbpS subunits and are located in close proximity to each other, over the monomer-monomer interface. Interestingly, Tyr77 is situated near to a postulated iron-binding site, containing Glu78 and Glu81 within an E-X-X-E motif (Figure 4) that has been previously characterized as an iron-binding motif [45–47].

The reported oxidative modifications in HbpS result in the degradation, either autonomously or protease dependent, of the oxidized protein [45]. A metal-catalyzed protein oxidation accompanied by cross-linking and degradation has been reported for the *Bradyrhizobium japonicum *iron response regulator (Irr) protein, which is involved in the regulation of iron transport as well as of heme biosynthesis genes [15]. It is proposed that the iron-catalyzed oxidation with subsequent degradation of Irr is the molecular basis for the modulation of the activity of this regulator.

The membrane-embedded sensor kinase PmrB from the two-component system PmrA-PmrB of the pathogen *Salmonella enterica* serovar typhimurium exhibits two copies of the EXXE iron-binding motif. These are periplasmically located and have been proposed to be involved in sensing of extracellular ferric iron. Upon iron sensing and autophosphorylation, PmrB transfers the signal to its cognate response regulator PmrA, which in turn regulates Fe^3+^ resistance genes: *pbgP* and *ugd* both coding for enzymes that modify the lipid A region of lipopolysaccharide (LPS) with 4-aminoarabinose. The gene product of *pmrC* catalyzes the addition of phosphoethanolamine to lipid A, whereas the phosphatase encoded by *pmrG* targets the phosphate located in the core region of the LPS. These modifications result in a less-charged cell surface and diminished binding of ferric iron to the membrane [48]. In contrast to other iron-sensing systems (i.e., PerR and HbpS-SenS-SenR), PmrA-PmrB seems to protect the cell against iron toxicity independent of the simultaneous presence of oxygen.

Beside the posttranslational modifications (i.e., oxidation of histidines, carbonylation, dityrosine formation, or S–S bonding) that regulate iron-sensing proteins, posttranscriptional regulation of gene expression in response to oxidative stress has been reported for the aconitases AcnA and AcnB of *Escherichia coli *[49, 50]. In their holo form, both AcnA and AcnB contain an iron-sulfur cluster, [4Fe-4S], and exhibit enzymatic function. Under reducing conditions, they catalyze the isomerisation of citrate to isocitrate. Upon oxidative stress and iron depletion, the iron-sulfur cluster is released. The resulting apo-AcnA and apo-AcnB proteins act as RNA-binding proteins that stabilize *acnA*- and *acnB*-mRNA transcripts, leading to increased amount of the respective aconitases that subsequently complex iron-sulfur clusters [49]. Moreover, mutational analyses have revealed that the presence of AcnA provides *E. coli* with enhanced resistance against superoxide-mediated oxidative stress. Additionally, proteomics studies demonstrated that the expression of anti-oxidative stress-working proteins (i.e., SodA and TrxB) is dependent on AcnA. 

## 4. Summary

As in humans, the exposure of bacteria to ROS causes damage to a variety of macromolecules, resulting in mutations and often in cell death. However, ROS may also be considered to be beneficial compounds, as they function as signalling molecules that lead to a coordinated response of bacteria under redox-stress conditions. These signals can be sensed by redox-active and zinc-coordinating Cys-X-X-Cys centres in proteins. Under reducing conditions, zinc stabilizes protein structure, but the presence of H_2_O_2_ provokes the release of zinc and the formation of S–S bridges that significantly alter the conformation and structure of the protein (i.e., FurS or RsrA). As a result, transcriptional activity is altered (by FurS), or the ability to interact with the partner DNA-binding protein is lost (by RsrA). In both cases, the transcription of genes involved in the anti-oxidative stress response is ultimately activated. The structural basis for the redox sensitivity is given by the location of S–S-forming cysteines in the protein, namely, their solvent exposition within the three-dimensional structure. Importantly, reduction and oxidation processes within Cys-X-X-Cys redox centres are reversible and provide bacteria with an elegant switch on/off mechanism.

Iron-dependent oxidative modifications (i.e., 2-oxo-histidine or dityrosine formation) are also involved in ROS-based signalling. Under H_2_O_2_-based stress, iron catalyzes the oxidation of histidines in the peroxide resistance regulator PerR, leading to release of iron and subsequently to de-repression, and expression of target genes. In analogy, iron-based stress activates the HbpS-SenS-SenR-mediated signalling cascade. HbpS is a redox sensor that upon oxidative modifications undergoes conformational and structural changes, inducing the phosphorylation cascade between the sensor kinase SenS and the response regulator SenR. As known for other regulators, oxidative modifications lead to the building of covalent bonds within proteins. Such processes are irreversible and lead ultimately to degradation of oxidized proteins. Therefore, *de novo* protein biosynthesis is required to switch off the corresponding signal cascade. 

The diversity of responsive protein elements among bacteria correlates with the diversity in: ecological niches, life cycles, pathogenic, and nonpathogenic character. Future efforts will undoubtedly demonstrate that additional further specialized systems exist.

## Figures and Tables

**Table 1:** Examples of proteins containing the redox-sensing motif Cys-X-X-Cys. (*) The references given are for the protein from the strain listed. Sequence alignments demonstrate that highly homologous proteins are found across many different bacterial species.

	Name	Strain*	Function	Zn^2+^-binding
Enzymatic function	Hsp33	*Escherichia coli *	The redox-regulated heat shock protein Hsp33 is a dual stress sensor responding to peroxide stress and increased temperature. Stress-mediated conformational changes result in zinc release and activation of Hsp33 chaperone function leading to suppression of protein aggregation [18].	Yes
	Trx2	*Escherichia coli*	Trx2 reductase activity is controlled by a redox switch within two CXXC motifs involved in zinc binding. Release of the bound zinc ion results in a conformational change leading to the reductase activity [23].	Yes
	ResA	*Bacillus subtilis*	Substrate selection of the membrane-bound thiol-disulfide oxidoreductase ResA is regulated by conformational changes determined by CXXC reduction or oxidation [24].	No
	DsbA	*Escherichia coli*	DsbA is a periplasmic protein oxidant for disulfide formation of extracellular proteins belonging to the Dsb family [25].	No
	SbcC	*Bacillus subtilis*	SbcC is a DNA repair protein with exonuclease activity [27].	Yes
	Cytochrome c	*Bacillus subtilis*	The covalent coordination of heme to apo-cytochrome c requires a reduced CXXC motif within the heme-binding motif [28].	No
	AhpF/AhpC	*Salmonella typhimurium*	AhpC and flavoprotein AhpF catalyze the pyridine nucleotide-dependent reduction of hydroperoxide substrates. AhpC, the peroxide-reducing component, is a scavenger of hydrogen peroxide in bacteria, whereas the disulfide reductase protein AhpF regenerates AhpC [29].	No
	CopA	*Thermotoga maritima*	CopA, a copper transport ATPase, sustains important roles in homeostasis of heavy metals and delivery of copper to metalloenzymes [30].	No
	HypA	*Escherichia coli*	HypA is required for nickel insertion into the hydrogenase precursor proteins [31].	No

Transcriptional regulator	FurS	*Streptomyces reticuli*	Oxidation of the transcriptional repressor FurS leads to derepression of the transcription of the gene *cpeB* coding for a catalase peroxidase [6].	Yes
	CatR	*Streptomyces coelicolor *A3 (2)	During peroxide stress, the Fur-like regulator CatR activates transcription of *catA* coding for catalase A [21].	No
	WhiB3	*Mycobacterium tuberculosis*	WhiB3 DNA binding to control the expression of genes coding for polyketide synthases is reversibly regulated by a thiol-disulfide redox switch. Reduction of the apo-WhiB3 Cys thiols of the CXXC motif suppresses genes regulating the synthesis of complex lipids, whereas oxidation stimulates it [22].	No
	SoxR	*Escherichia coli*	SoxR senses superoxide stress through a CXXC-coordinated [2Fe-2S]-cluster that results in transcriptional activation of a superoxide response regulon [32].	No
	SurR	*Pyrococcus furiosus (Archaea)*	A redox switch regulates the transcriptional regulator SurR. Oxidation with S^0^ inhibits DNA binding by SurR, leading to repression of genes related to H_2_ production and activation of genes involved in S^0^ metabolism [33].	No

Regulatory element	Spx	*Bacillus subtilis*	Global oxidative stress regulator interacting with the *α*-subunit of RNA polymerase for transcriptional induction of genes involved in thiol homeostasis (*mrsA-mrsB* operon) [19].	No
	RsrA	*Streptomyces coelicolor *A3 (2)	Antisigma factor RsrA negatively regulates expression of the thioredoxin system in response to cytoplasmatic oxidative stress. Under reducing conditions, RsrA binds to *σ* ^R^ resulting in inhibition of transcription [20].	Yes
	RslA	*Mycobacterium tuberculosis*	Membrane-associated RslA oxidation results in the release of bound Zn^2+^ through disulfide bond formation within the CXXC motif. The resulting conformational change leads to decreased *σ* ^L^ binding. The released sigma factor regulates the expression of genes involved in cell-wall and polyketide synthesis [17].	Yes
	RshA	*Mycobacterium tuberculosis*	RshA is an antisigma factor of the central regulator SigH that responds to oxidative and heat stress; it functions as a negative regulator of the alternative sigma factor SigH activity under reducing conditions [26].	No

**Table 2:** Examples of iron-dependent redox sensor proteins in bacteria. (*) The references given are for the protein from the strain listed. Sequence alignments demonstrate that highly homologous proteins are found across many different bacterial species.

	Name	Strain*	Function
Transcriptional regulator	Fur	*Escherichia coli*	Regulator with iron-dependent DNA-binding affinity negatively regulates genes involved in ferric iron uptake [38].
	DtxR	*Corynebacterium glutamicum*	DtxR acts as a global iron-mediated regulator, activating genes involved in iron storage and DNA protection and repressing genes involved in iron uptake and utilization [39].
	RirA	*Rhizobium leguminosarum*	Transcriptional regulator RirA is involved in ferric uptake regulation by regulating genes coding for iron transport, siderophore biosynthesis, and iron-sulfur cluster assembly [40].
	Irr	*Bradyrhizobium japonicum*	Iron response regulator (Irr) senses iron through the status of heme biosynthesis to regulate gene expression involved in iron homeostasis [42].
	PerR	*Bacillus subtilis*	DNA binding by the regulator PerR in response to peroxide stress is iron dependent [51].
	DmdR1	*Streptomyces coelicolor*	The transcriptional regulator DmdR1 regulates genes involved in desferrioxamine production in response to iron availability [43].
	IdeR	*Mycobacterium smegmatis*	IdeR negatively regulates siderophore biosynthesis involved in iron acquisition [44].
	SirR	*Staphylococcus epidermidis*	SirR is a Fe^2+^or Mn^2+^-dependent transcriptional repressor regulating the *sit*ABC operon encoding an ATPase, a cytoplasmic membrane protein, and the 32-kDa lipoprotein involved in siderophore-mediated iron uptake [45].
	FNR	*Escherichia coli*	Transcription factor FNR regulates gene expression in response to oxygen deficiency by its redox-sensitive bound iron. Binding of an iron-sulfur cluster is required for a conformational change to enhance DNA binding [46].
	IscR	*Escherichia coli*	[2Fe-2S]-cluster assembly regulates activity in transcription factor IscR of genes coding for proteins involved in iron-sulfur cluster assembly [47].
	TroR	*Treponema denticola*	TroR is a Mn^2+^ and Fe^2+^-dependent repressor of the ATP-binding cassette cation transport system (*tro*ABCD) regulating manganese and iron homeostasis [50].

Regulatory element	HbpS/SenS/SenR	*Streptomyces reticuli*	Iron-dependent activation/inhibition of the two-component system SenS-SenR involved in oxidative stress response through heme degradation and associated secondary structural changes [8].
	PmrA/PmrB	*Salmonella enterica*	The PmrA/PmrB two-component system senses iron and regulates the transcription of genes involved in iron resistance [41].
	ChrS/ChrA	*Corynebacterium diphteriae*	ChrS, the heme-sensing sensor kinase of the two-component system ChrS/ChrA, regulates genes involved in utilization of host heme as an iron source and in protecting the bacteria against the toxic effects of heme [48].
	FecA/FecR/FecI	*Escherichia coli*	The periplasmic protein FecR senses periplasmic iron dicitrate by the outer membrane protein FecA which is loaded with ferric citrate. FecR transmits the signal to the sigma factor FecI which results in transcriptional activation of the *fec*-operon for ferric citrate transport [52].
	AcnA/AcnB	*Escherichia coli*	The aconitases AcnA (induced by iron and oxidative stress) and AcnB posttranscriptionally regulate gene expression (i.e., *sodA*) by an iron-sulfur cluster-dependent switch [49].
